# Linkage to HIV Care Among Adolescents and Young Adults Reporting a Positive HIV Self-Test in OR Tambo District, Eastern Cape, South Africa

**DOI:** 10.3390/epidemiologia7050138

**Published:** 2026-10-07

**Authors:** Zimkhitha Diniso, Guillermo Alfredo Pulido Estrada, Sibusiso Cyprian Nomatshila, Kululwa Ndayi, Monwabisi Faleni

**Affiliations:** 1 School of Public Health, Faculty of Medicine and Health Sciences, Walter Sisulu University, Mthatha 5100, South Africa; 214168646@mywsu.ac.za (Z.D.); gpulidoestrada@wsu.ac.za (G.A.P.E.); snomatshila@wsu.ac.za (S.C.N.); kndayi@wsu.ac.za (K.N.); 2 WSU Society and Health Research Institute, Walter Sisulu University, Mthatha 5100, South Africa

**Keywords:** HIV self-testing, adolescents, young adults, linkage to care, post-HIVST outcomes, confirmatory testing

## Abstract

Background: HIV self-testing (HIVST) can improve access to HIV testing among adolescents and young adults (AYAs); however, gaps remain in post-test linkage to care. This study examined post-HIVST outcomes and factors associated with reported linkage to HIV care among AYAs in the Eastern Cape, South Africa. Methods: A facility-based cross-sectional study was conducted among 170 AYAs aged 15–24 years who had used HIVST within the preceding five months. Socio-demographic characteristics, HIVST results, post-test care-seeking, and linkage to HIV care were assessed. Bivariate analyses and Firth penalized logistic regression were used to identify factors associated with reported linkage to HIV care among participants reporting a positive HIVST result. Results: Of 156 participants who disclosed their HIVST result, 67 (42.9%) reported a positive result. Among these, 29 (43.3%) sought confirmatory testing, 38 (56.7%) sought care within 1–4 weeks, and 53 (79.1%) reported linkage to HIV care. More than half (55.2%) reported that counselling was not offered, and 59.7% perceived linkage-to-care services as not easily accessible. In multivariable analysis, female participants had higher odds of reporting linkage to HIV care than male participants (AOR = 5.54, 95% CI: 1.49–20.67, *p* = 0.011). Conclusions: Among AYAs with previous HIVST use, important gaps were observed in confirmatory testing and timely care-seeking. Male AYAs may require targeted support to improve engagement with HIV care services.

## 1. Introduction

The global HIV epidemic continues to disproportionately affect adolescents and young adults (AYAs) aged 15–24 years, who account for approximately one-third of all new HIV infections worldwide [1]. In sub-Saharan Africa, which bears the highest burden of HIV, young people contribute 35–40% of new infections in countries such as South Africa, Mozambique, and Eswatini [2]. Despite significant advances in HIV prevention and treatment, AYAs consistently lag behind adults across the HIV care continuum, with lower rates of status awareness, antiretroviral therapy (ART) initiation, and viral suppression [3]. The UNAIDS 95-95-95 targets, aiming for 95% of people living with HIV knowing their status, 95% of those on treatment, and 95% of those virally suppressed by 2025, remain elusive for this priority population [4].

HIV self-testing (HIVST) has been heralded as a game-changing innovation to address testing gaps among hard-to-reach and underserved populations [5]. Since the World Health Organization (WHO) issued its first recommendations on HIVST in 2016, over 90 countries have adopted policies supporting self-testing, with more than 30 million HIVST kits distributed in sub-Saharan Africa alone [6]. The HIVST offers several advantages for AYAs: privacy, convenience, autonomy, and the ability to circumvent facility-based barriers such as stigma, discrimination, and inconvenient clinic hours [7]. A recent systematic review and meta-analysis demonstrated that 79% of AYAs who were offered HIVST completed the test, with 62% being first-time testers, confirming HIVST’s potential to reach previously untested youth [8].

However, the promise of HIVST extends beyond testing uptake to encompass the full cascade of care following a positive result. Linkage to confirmatory testing and subsequent ART initiation represents the critical bridge between diagnosis and treatment that determines HIVST’s public health impact [9]. Emerging evidence suggests that while HIVST substantially increases testing coverage, linkage to care following positive self-tests remains suboptimal. A recent systematic review of programmes in Southern Africa reported substantially lower linkage to care following a positive result in HIVST programmes compared with clinician-administered testing programmes (pooled OR = 0.036, 95% CI: 0.025–0.051); however, the authors cautioned that this programme-level comparison was limited by ecological differences and heterogeneity in the measurement of linkage outcomes. Similarly, a multi-country analysis revealed that only 43–60% of individuals with reactive HIVST results successfully linked to confirmatory testing and care [10].

Among AYAs, linkage challenges may be particularly pronounced due to developmental, structural, and psychosocial factors. Young people face unique barriers, including fear of disclosure, limited health literacy, mobility, transportation costs, and health systems that are often not youth friendly [11]. Gender dynamics further complicate the picture, with evidence indicating that male AYAs are less likely than their female counterparts to engage with health services following HIV diagnosis [12]. In Mozambique, a population-based survey found that 58% of HIV-positive young people were out-of-care, with males and those in Northern provinces at greatest risk [13]. Understanding the post-HIVST outcomes and their determinants among AYAs is essential for designing effective interventions to improve linkage outcomes. Despite the rapid scale-up of HIVST across sub-Saharan Africa, there remains a paucity of granular data on post-HIVST outcomes, including confirmatory testing, treatment initiation, and retention among young people [14]. Existing studies have primarily focused on testing uptake and acceptability, with less attention to the “second 95” of the UNAIDS targets [15,16]. This study aimed to address this gap by comprehensively examining the post-HIVST outcomes among AYAs in OR Tambo District, Eastern Cape, South Africa. Specifically, the study aimed to: (1) describe the socio-demographic characteristics of AYAs who had used HIVST; (2) describe the self-reported post-HIVST outcomes among participants reporting a positive HIV self-test result, including confirmatory testing, care-seeking, and linkage to HIV care; (3) assess post-test support, counselling, and perceived accessibility of linkage-to-care services; and (4) identify factors associated with reported linkage to HIV care among participants reporting a positive HIVST result. The findings are intended to inform interventions aimed at strengthening post-HIVST support and linkage to HIV care among AYAs.

## 2. Materials and Methods

### 2.1. Study Design and Setting

This facility-based cross-sectional study was conducted between July 2025 and November 2025 in the OR Tambo district, Eastern Cape, South Africa. The Eastern Cape has a generalized HIV epidemic with prevalence among AYAs estimated at 9.6%, and HIVST was introduced in South Africa in 2016 as part of the national HIV testing policy, with pilot implementation between 2017 and 2019. In the Eastern Cape, the wider programmatic rollout and facility-based implementation accelerated around 2020 to date through public-sector distribution, facility-based programmes, and private-sector channels. Participant recruitment was conducted at three health facilities in OR Tambo District: Gateway Clinic and Ngangelizwe Community Health Centre, both located in urban areas, and Maxwele Clinic, located in a rural area. The three facilities were purposively selected for participant recruitment based on their convenience and accessibility.

### 2.2. Study Population and Sampling

Eligible participants were adolescents and young adults aged 15–24 years who attended the selected health facilities during the study period and reported having used HIV self-testing within the five months preceding data collection. Eligible participants were recruited using consecutive sampling and were approached as they attended the selected facilities until the required sample size was reached. Previous HIV diagnosis was not specified as an exclusion criterion; therefore, participants with a previous HIV diagnosis could have been eligible if they otherwise met the study eligibility criteria. Exclusion criteria were: (1) age < 15 or >24 years; (2) inability to provide informed consent or, for participants aged 15–17 years, parental consent and adolescent assent; (3) never having used HIVST; and (4) severe cognitive impairment or acute illness precluding participation. Information on previous HIV diagnosis, ART use, and Pre-exposure Prophylaxis (PrEP) use was not collected in this study. The sample size was calculated using the single-population proportion formula (n = Z^2^p(1 − p)/d^2^), assuming a 50% proportion to provide the maximum required sample size, a 95% confidence level, and an 8% margin of error. This yielded a minimum sample size of approximately 150 participants. After adjusting for a 10% contingency for incomplete responses, the required sample size was approximately 167 participants, which was rounded upward to a target of 170 participants.

### 2.3. Data Collection

Trained research assistants administered a structured questionnaire through face-to-face interviews in private settings at the selected health facilities. The questionnaire comprised 50 closed-ended questions organized into five sections: socio-demographic characteristics; awareness of HIVST; HIVST use and experiences; HIVST results and linkage to care; and perceptions and attitudes toward HIVST. The questionnaire was pilot-tested with 15 adolescents and AYAs prior to the main data collection. Socio-demographic information included age, gender, educational level, employment status, relationship status, place of residence, household income, access to healthcare facilities, religion, and living arrangements. The HIVST related information included awareness and knowledge of HIVST, previous use and frequency of HIVST, source of self-test kits, experiences with self-testing, and the most recent HIVST result. For participants reporting a positive HIV self-test result, post-test outcomes included confirmatory testing, time to seek care, facility attended for confirmatory testing, linkage to HIV care, post-test support, counselling, and perceived accessibility of linkage-to-care services. The questionnaire evaluated participants’ perceptions and attitudes toward HIV self-testing (HIVST). The outcomes related to post-HIVST were gathered through distinct self-reported measures. Confirmatory testing was assessed by asking participants, “Did you seek confirmatory testing after a positive self-test?” Care-seeking behavior was evaluated separately by asking, “How soon did you seek care after testing positive?” The response options included within one week, 1–4 weeks, after one month, or did not seek care. Linkage to HIV care was assessed by asking, “Were you linked to HIV care services after a positive result?” and was treated as a self-reported binary outcome (yes/no). The questionnaire did not further define linkage in terms of attendance at an HIV care visit, enrolment in HIV care, or ART initiation. These variables were treated as analytically distinct measures. The questionnaire did not establish that confirmatory testing, care-seeking, and linkage to care necessarily occurred as sequential events; therefore, reporting no confirmatory testing was not assumed to indicate that no other form of care had been sought. Post-test support included satisfaction with post-test support (very satisfied, satisfied, dissatisfied, or very dissatisfied), counselling services offered (yes/no), and perceived accessibility of linkage-to-care services (easy/not easy). An HIVST acceptability composite index was calculated from questionnaire items assessing participants’ experiences and perceptions of HIV self-testing. Items were coded so that higher scores represented greater acceptability and were summed to generate a composite score. The composite score was dichotomized using the median score of three, with scores greater than three classified as low acceptability, and scores less than or equal to three classified as high acceptability.

### 2.4. Ethical Considerations

The study received ethical approval from the Walter Sisulu University Health Sciences Research Ethics Committee, with reference number WSU HREC 092/2025, 27 June 2025. All participants provided written informed consent (with parental consent and adolescent assent for participants aged 15–17 years). Participants were assured of confidentiality, anonymity in reporting, and the right to withdraw without consequence. Those reporting positive HIVST results who were not linked to care were offered facilitated referral to confirmatory testing and treatment services.

### 2.5. Statistical Analysis

Data analysis was performed using SPSS version 30.0 for descriptive and bivariate analyses, while Stata version 19 was used for the multivariable analysis. Descriptive statistics summarized socio-demographic characteristics and post-HIVST outcomes, presented as frequencies and percentages. For gender analyses, participants selecting “prefer not to say” were excluded from gender-specific calculations. Bivariate analysis examined associations between independent variables (socio-demographic characteristics, HIVST frequency, confirmatory testing, time to care, counselling, perceived accessibility, acceptability) and the primary outcome (linkage to HIV care). Chi-square tests were used for categorical variables; Fisher’s exact test was applied where expected cell counts were <5. Statistical significance was set at *p* < 0.05.

Variables with *p* < 0.20 in bivariate analysis were considered for inclusion in the multivariable analysis. Age group, gender, employment status, and place of residence were included in the multivariable model. Although time to seek care was associated with reported linkage in the bivariate analysis, it was not included in the multivariable model because its temporal relationship with reported linkage to HIV care was not established. Because of the small number of non-linkage events (14 of 67 participants with a positive HIV self-test result), Firth penalized logistic regression was used to reduce small-sample bias and improve the stability of parameter estimates. The final multivariable model included age group, gender, employment status, and place of residence. One participant with missing gender data was excluded from the multivariable analysis, resulting in an analytical sample of 66 participants. Adjusted odds ratios (AOR) with 95% confidence intervals (CI) were calculated. Statistical significance in the multivariable analysis was set at *p* < 0.05.

## 3. Results

### 3.1. Socio-Demographic Characteristics of Participants

A total of 170 adolescents and young adults participated in the study. Table 1 presents the socio-demographic characteristics. The majority of participants (58.2%) were aged 20–24 years, while 41.8% were in the younger adolescent group (15–19 years). After excluding two participants who preferred not to disclose their gender, 64.3% were female, and 35.7% were male.

Educational attainment was relatively high, with nearly half of participants (48.2%) having completed secondary education and 41.2% having attained tertiary education. Slightly more than half (51.2%) were employed, while 48.8% were students. Regarding marital status, most participants were in a relationship (58.2%), followed by those who were single (34.1%), while a smaller proportion were married (7.6%). The majority (61.8%) resided in rural areas, while 38.2% lived in urban areas.

### 3.2. HIV Self-Test Results

Table 2 displays participants’ most recent HIV self-test results. Of the 170 participants, 14 (8.2%) selected “prefer not to say” regarding their test result and were excluded from percentage calculations. Among the 156 participants who disclosed their results, the majority (57.1%) reported a negative HIV self-test result, while 42.9% reported a positive result.

**Figure 1:**
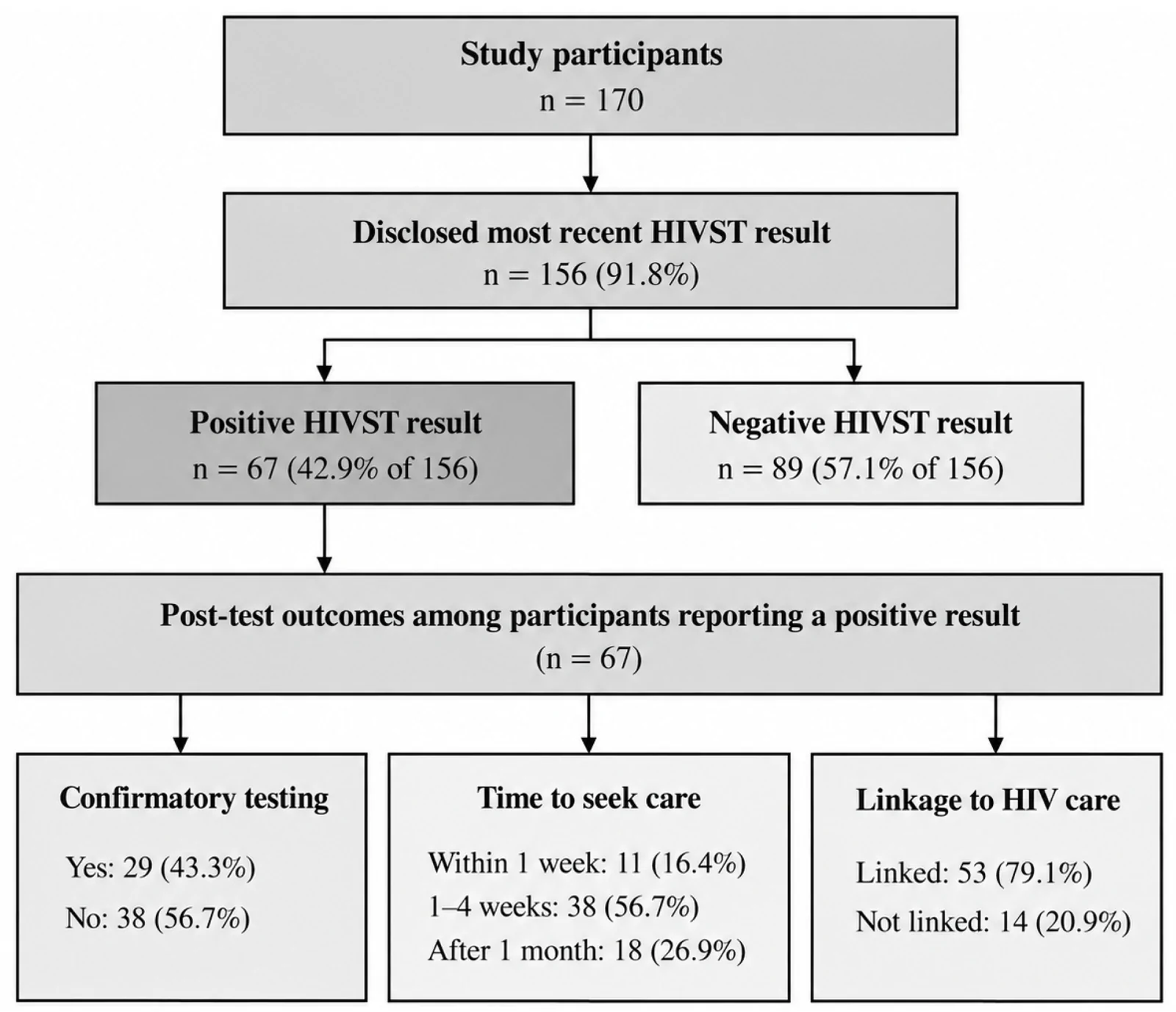
HIV self-testing results and post-test outcomes among adolescents and young adults. Note: Confirmatory testing, time to seek care, and linkage to HIV care were assessed as separate self-reported outcomes and should not be interpreted as verified sequential stages.

Of the 170 participants enrolled in the study, 156 (91.8%) disclosed their most recent HIVST result. Figure 1 summarizes the distribution of HIVST results and the post-test outcomes among participants reporting a positive result, including confirmatory testing, time to seek care, and linkage to HIV care.

### 3.3. Post-HIV Self-Testing Outcomes

Table 3 presents the post-HIVST outcomes among the 67 participants who reported a positive HIV self-test result. Less than half (43.3%) sought confirmatory testing after a positive self-test, while the majority (56.7%) did not.

Regarding timeliness of care-seeking, only 16.4% sought care within one week of testing positive, while more than half (56.7%) sought care between one and four weeks post-test. A notable proportion (26.9%) delayed seeking care for more than 1 month. Among participants who sought confirmatory testing (n = 29), private doctors were the most commonly reported providers (48.3%), followed by clinics (37.9%) and hospitals (13.8%).

Among participants reporting a positive HIV self-test result, 79.1% reported being linked to HIV care services, while 20.9% reported no linkage to care. The higher proportion reporting linkage to HIV care than to confirmatory testing should be interpreted with caution, as confirmatory testing and linkage were assessed as separate self-reported measures and were not independently verified through clinical records.

Post-test support indicators revealed substantial gaps. While 68.6% reported being satisfied or very satisfied with support received, nearly one-third (31.4%) expressed dissatisfaction. Critically, counselling services were offered to less than half of participants (44.8%), and the majority (59.7%) perceived linkage-to-care services as not easily accessible.

### 3.4. Factors Associated with Linkage to HIV Care: Bivariate Analysis

Table 4 shows the factors associated with reported linkage to HIV care among participants reporting a positive HIV self-test result. Gender and time to seek care were significantly associated with linkage to HIV care in the bivariate analysis. Female participants had a significantly higher proportion of linkage to HIV care than males (89.1% vs. 60.0%, *p* = 0.015). Participants who sought care early (≤4 weeks) had a higher proportion of linkage than those who delayed seeking care for more than one month (87.8% vs. 55.6%, *p* = 0.007). Relationship status was not significantly associated with linkage to HIV care (*p* = 0.792), with linkage reported by 81.3% of single participants, 80.0% of those in a relationship, and 66.7% of married participants. Age group, employment status, and place of residence had *p*-values <0.20 and, together with gender, were included in the multivariable analysis. Although time to seek care was associated with reported linkage in the bivariate analysis, it was not included in the multivariable model because the temporal sequence between these measures was not established.

### 3.5. Multivariable Analysis of Factors Associated with Reported Linkage to HIV Care

The Firth penalized logistic regression model included age group, gender, employment status, and place of residence (Table 5). The overall model was statistically significant (Wald χ^2^(4) = 9.61, *p* = 0.048). Female participants had significantly higher adjusted odds of reporting linkage to HIV care compared with male participants (AOR = 5.54, 95% CI: 1.49–20.67, *p* = 0.011). Age group (AOR = 0.42, 95% CI: 0.10–1.75, *p* = 0.235), employment status (AOR = 0.28, 95% CI: 0.07–1.10, *p* = 0.069), and place of residence (AOR = 1.66, 95% CI: 0.45–6.13, *p* = 0.445) were not statistically significantly associated with reported linkage to HIV care.

## 4. Discussion

This study examined self-reported post-HIVST outcomes among adolescents and young adults (AYAs) and identified important gaps in confirmatory testing, timely care-seeking, and post-test support. Among AYAs with previous HIVST use included in this study, gaps were observed across the separately assessed post-HIVST outcomes, including confirmatory testing, care-seeking, and reported linkage to HIV care. Female gender was associated with higher adjusted odds of reported linkage to HIV care compared with male gender.

The proportion of participants reporting a positive HIV self-test result (42.9%) was substantially higher than the estimated HIV prevalence of 9.6% among AYAs in the region. However, this proportion should not be interpreted as an estimate of HIV prevalence or HIVST coverage in the wider AYA population. The study was facility-based and included only AYAs who had used HIVST within the preceding five months, resulting in a selected group that may differ systematically from the general AYA population. Furthermore, a previous HIV diagnosis was not an exclusion criterion, and information on previous HIV diagnosis and pre-existing ART use was not collected in this study. Consequently, it was not possible to distinguish participants with newly identified HIV infection from those with a previously known HIV-positive status. The facility-based recruitment strategy, restriction to previous HIVST users, and inability to distinguish newly identified from previously diagnosed HIV infections may therefore have contributed to the relatively high proportion of positive HIVST results and indicate the potential for selection bias. Accordingly, the findings should be interpreted as describing the study population rather than the prevalence of HIV or the coverage of HIVST among AYAs in the Eastern Cape.

A major concern was the low uptake of confirmatory testing, with only 43.3% of participants reporting confirmatory testing after a positive HIVST result. Similar challenges have been reported across sub-Saharan Africa, where linkage following HIVST remains lower than for facility-based testing [17,18,19,20]. Barriers may include stigma, transport costs, inconvenient clinic hours, confidentiality concerns, and health-system limitations [21,22,23]. Among participants who reported seeking confirmatory testing, private doctors were the most frequently reported testing venue (48.3%), followed by clinics (37.9%) and hospitals (13.8%). However, the study did not collect information on the costs of confirmatory testing, health insurance, or payment status; therefore, the reasons for participants’ choice of testing venue and its implications for financial access or equity could not be assessed.

Early linkage is associated with faster ART initiation, improved clinical outcomes, and reduced HIV transmission [24,25]. Delays may result from fear of disclosure, denial, limited understanding of treatment benefits, or logistical barriers such as transport costs and competing school or work commitments [26]. Facility-based confirmatory testing, peer navigation, and decentralized ART services may help address these barriers [27,28].

A notable finding was the significant gender disparity in linkage to care. Male AYAs had substantially lower odds of linkage than females, consistent with evidence showing poorer engagement of men across the HIV care cascade [29,30]. Factors such as masculine norms, fear of disclosure, work-related constraints, and limited male-friendly health services may contribute to this disparity [31,32]. These findings support the need for targeted interventions, including male-friendly service models, peer support programmes, workplace testing initiatives, and facility-based HIVST distribution strategies [33].

The study also identified substantial deficiencies in post-test support. More than half of participants reported receiving no counselling after HIVST, and most perceived linkage services as inaccessible. While HIVST improves privacy and autonomy, counselling remains essential for supporting individuals through diagnosis and linkage to care [6,34]. Digital counselling platforms and peer navigation programmes have shown promise in improving linkage among young people and may provide scalable solutions in resource-limited settings [35,36].

An important observation was the discrepancy between reported linkage to HIV care (79.1%) and confirmatory testing (43.3%). As confirmatory testing, care-seeking, and linkage to HIV care were assessed as separate self-reported outcomes, participants who reported not seeking confirmatory testing were not necessarily indicating that they had not sought other forms of care. Nevertheless, this discrepancy should be interpreted cautiously. Information on previous HIV diagnosis, pre-existing ART use, and PrEP use was not collected in this study; therefore, it was not possible to determine whether all participants reporting a positive HIV self-test represented newly identified HIV infections. Furthermore, linkage to care was not independently verified through clinical records, and the temporal sequence between the reported HIV self-test and linkage to care could not be confirmed. These limitations, together with potential differences in participants’ understanding of the post-test outcomes and possible recall or self-reporting bias, may have contributed to the observed discrepancy. Future HIVST studies should use clearly defined and standardized measures and prospectively document previous HIV diagnosis, ART and PrEP use, confirmatory testing, and subsequent linkage to care.

These findings have several implications for HIV programmes. HIVST initiatives should strengthen referral pathways for confirmatory testing, integrate counselling and peer support services, prioritize rapid post-test follow-up, and implement targeted interventions for male AYAs. Routine monitoring of confirmatory testing, linkage, and treatment initiation is also necessary to identify gaps and improve programme performance.

### 4.1. Strengths and Limitations

A key strength of this study is its comprehensive assessment of the post-HIVST outcomes among AYAs, including confirmatory testing, care-seeking behavior, counselling, and linkage outcomes. However, the cross-sectional design limits causal inference, and reliance on participants’ retrospective self-reports of HIVST experiences and post-test events during the five-month eligibility window may have introduced recall and social desirability bias. Fourteen participants (8.2%) did not disclose their most recent HIV self-test result and were excluded from analyses requiring HIVST result status. Because non-disclosure may be associated with factors such as stigma, HIV status, or healthcare engagement, their exclusion may have introduced selection bias and affected the estimated post-HIVST outcomes. Importantly, the multivariable analysis was restricted to the 67 participants who reported a positive HIV self-test result, of whom only 14 were not linked to HIV care. The relatively small number of outcome events in relation to the number of predictors included in the model limited statistical power and precision, as reflected by the wide confidence intervals around several adjusted odds ratios. Consequently, the multivariable estimates should be interpreted cautiously and regarded as exploratory rather than definitive. The sampling approach may also limit the generalizability of the findings to all AYAs using HIVST. In addition, linkage outcomes were not verified through clinical records, and qualitative exploration of barriers to care was not undertaken.

### 4.2. Future Research

Future studies should employ longitudinal designs to track individuals from HIVST through treatment initiation and retention in care. Research evaluating interventions that improve confirmatory testing, reduce delays in care-seeking, and address gender disparities is particularly needed. Qualitative studies exploring the experiences of male AYAs and those who fail to link to care would provide valuable insights for programme development.

## 5. Conclusions

HIV self-testing represents an important HIV testing approach for adolescents and young adults; however, important gaps remain in confirmatory testing, timely care-seeking, counselling, and linkage to care. Lower reported linkage among male than female participants highlights the importance of gender-responsive linkage support. Strengthening post-test support systems, improving access to confirmatory testing, implementing gender-responsive interventions, and routinely monitoring linkage outcomes are essential to maximize the contribution of HIVST towards achieving UNAIDS HIV targets and improving HIV outcomes among young people.

## Figures and Tables

**Table 1:** Socio-demographic characteristics of participants.

Variable	Category	n	%
Age group (years)	15–19	71	41.8
	20–24	99	58.2
Gender *	Male	60	35.7
	Female	108	64.3
Level of education	No formal education	2	1.2
	Primary school	16	9.4
	Secondary school (Grade 8–12)	82	48.2
	Tertiary (College/TVET)	70	41.2
Employment status	Employed	87	51.2
	Student	83	48.8
Marital status	Single	58	34.1
	In a relationship	99	58.2
	Married	13	7.6
Place of residence	Urban	65	38.2
	Rural	105	61.8

* Percentages for gender calculated excluding two participants who selected “Prefer not to say”.

**Table 2:** HIV self-test results among participants who used HIV self-test.

HIV Self-Test Result	n	%
Negative	89	57.1
Positive	67	42.9
Total	156	100

**Table 3:** Self-reported post-HIV self-test outcomes among participants reporting a positive HIV self-test result (n = 67).

Variable	Category	n	%
Sought confirmatory testing after a positive HIV self-test	Yes	29	43.3
	No	38	56.7
Time to seek care after testing positive	Within 1 week	11	16.4
	1–4 weeks	38	56.7
	After 1 month	18	26.9
Facility attended for confirmatory testing *	Clinic	11	37.9
	Private doctor	14	48.3
	Hospital	4	13.8
Reported linkage to HIV care	Yes	53	79.1
	No	14	20.9
Satisfaction with post-test support	Very satisfied	8	11.9
	Satisfied	38	56.7
	Dissatisfied	18	26.9
	Very dissatisfied	3	4.5
Counselling services offered after HIV self-test	Yes	30	44.8
	No	37	55.2
Perceived accessibility of linkage-to-care services	Yes	27	40.3
	No	40	59.7

Note: * Percentages for facility attended for confirmatory testing are calculated among participants who reported seeking confirmatory testing (n = 29). All other percentages are based on participants with a positive HIV self-test result (n = 67).

**Table 4:** Factors associated with reported linkage to HIV care among participants reporting a positive HIV self-test result (n = 67).

Variable	Category	Linked to HIV Care n (%)	Not Linked to HIV Care n (%)	*p*-Value
Age group (years)	15–19	22 (88.0)	3 (12.0)	0.167
	20–24	31 (73.8)	11 (26.2)	
Gender **	Male	12 (60.0)	8 (40.0)	0.015 *
	Female	41 (89.1)	5 (10.9)	
Marital status	Single	13 (81.3)	3 (18.8)	0.792 *
	In a relationship	36 (80.0)	9 (20.0)	
	Married	4 (66.7)	2 (33.3)	
Level of education	≤Primary	8 (80.0)	2 (20.0)	0.594 *
	Secondary	26 (81.3)	6 (18.8)	
	Tertiary	19 (76.0)	6 (24.0)	
Employment status	Employed	30 (85.7)	5 (14.3)	0.164
	Student	23 (71.9)	9 (28.1)	
Place of residence	Urban	20 (71.4)	8 (28.6)	0.190
	Rural	33 (84.6)	6 (15.4)	
HIV self-test use frequency	Once	11 (84.6)	2 (15.4)	0.652 *
	≥2 times	42 (77.8)	12 (22.2)	
Sought confirmatory testing	Yes	21 (72.4)	8 (27.6)	0.239
	No	32 (84.2)	6 (15.8)	
Time to seek care	Early (≤4 weeks)	43 (87.8)	6 (12.2)	0.007 *
	Delayed (>1 month)	10 (55.6)	8 (44.4)	
Counselling offered after HIV self-test	Yes	22 (73.3)	8 (26.7)	0.295
	No	31 (83.8)	6 (16.2)	
Perceived accessibility of services	Easy	20 (74.1)	7 (25.9)	0.405
	Not easy	33 (82.5)	7 (17.5)	
HIV self-test acceptability level	Low	16 (80.0)	4 (20.0)	0.906
	High	37 (78.7)	10 (21.3)	

* Fisher’s exact test applied where expected cell counts were <5. ** One participant who selected “prefer not to say” for gender was excluded from the gender-specific analysis; therefore, the total for gender is n = 66.

**Table 5:** Factors associated with reported linkage to HIV care among adolescents and young adults with a positive HIV self-test result: Firth penalized logistic regression.

Variable	Category (Reference)	AOR	95% CI	*p*-Value
Age group	20–24 vs. 15–19	0.42	0.10–1.75	0.235
Gender	Female vs. Male	5.54	1.49–20.67	0.011
Employment status	Student vs. Employed	0.28	0.07–1.10	0.069
Place of residence	Rural vs. Urban	1.66	0.45–6.13	0.445

Note: AOR = adjusted odds ratio; CI = confidence interval. Estimates were obtained using Firth penalized logistic regression. Reference categories are shown after “vs.”.

## Data Availability

The datasets used are available from the corresponding author upon reasonable request.

## Abbreviations

The following abbreviations are used in this manuscript:

HIV	Human Immunodeficiency Virus
HIVST	Human Immunodeficiency Virus Self-Testing
AYAs	Adolescents and Young Adults
WHO	World Health Organization
